# Predictive Factors for Reintubation following Noninvasive Ventilation in Patients with Respiratory Complications after Living Donor Liver Transplantation

**DOI:** 10.1371/journal.pone.0081417

**Published:** 2013-12-05

**Authors:** Yuichi Chihara, Hiroto Egawa, Toru Oga, Tomomasa Tsuboi, Tomohiro Handa, Shintaro Yagi, Taku Iida, Atsushi Yoshizawa, Kazuhiko Yamamoto, Michiaki Mishima, Koichi Tanaka, Shinji Uemoto, Kazuo Chin

**Affiliations:** 1 Department of Respiratory Medicine, Kyoto University Hospital, Kyoto, Japan; 2 Department of Gastroenterological Surgery, Tokyo Women's Medical University Hospital, Tokyo, Japan; 3 Departments of Respiratory Care and Sleep Control Medicine, Kyoto University Graduate School of Medicine, Kyoto, Japan; 4 Department of Rehabilitation, Kyoto University Hospital, Kyoto, Japan; 5 Department of Transplant Surgery, Kyoto University Hospital, Kyoto, Japan; 6 Departments of Allergy and Rheumatology, Tokyo University Graduate School of Medicine, Tokyo, Japan; 7 Foundation for Kobe International Medical Alliance, Kobe, Japan; San Raffaele Scientific Institute, Italy

## Abstract

**Background:**

Postoperative respiratory complications are a major cause of mortality following liver transplantation (LT). Noninvasive ventilation (NIV) appears to be effective for respiratory complications in patients undergoing solid organ transplantation; however, mortality has been high in patients who experienced reintubation in spite of NIV therapy. The predictors of reintubation following NIV therapy after LT are not exactly known.

**Methods:**

Of 511 adult patients who received living-donor LT, data on the 179 who were treated by NIV were retrospectively examined.

**Results:**

Forty-three (24%) of the 179 patients who received NIV treatment required reintubation. Independent factors associated with reintubation by multivariate logistic regression analysis were controlled preoperative infections (odds ratio [OR] 8.88; 95% confidence interval (CI) 1.64 to 48.11; p = 0.01), ABO-incompatibility (OR 4.49; 95% CI, 1.50 to 13.38; p = 0.007), and presence of postoperative pneumonia at the time of starting NIV (OR 3.28; 95% CI, 1.02 to 11.01; p = 0.04). The reintubated patients had a significant higher rate of postoperative infectious complications and a significantly longer intensive care unit stay than those in whom NIV was successful (p<0.0001). Of the 43 reintubated patients, 22 (51.2%) died during hospitalization following LT vs. 8 (5.9%) of the 136 patients in whom NIV was successful (p<0.0001).

**Conclusions:**

Because controlled preoperative infection, ABO-incompatibility or pneumonia prior to the start of NIV were independent risk factors for reintubation following NIV, caution should be used in applying NIV in patients with these conditions considering the high rate of mortality in patients requiring reintubation following NIV.

## Introduction

Liver transplantation (LT) has become the mainstay for the treatment of end-stage liver disease, acute liver failure, hepatocellular cancer, and some metabolic liver diseases [1]. Liver transplantation in Japan is highly dependent on living donors because of a severe deficiency in the availability of liver grafts from deceased donors [2].

Postoperative respiratory complications (PRCs) such as atelectasis, pleural effusion, pulmonary edema, and pneumonia are frequent after LT and their incidence is reported to be between 44% and 87% [3]–[8]. Furthermore, persistent pulmonary edema, pleural effusion, and atelectasis have been reported to be major independent predictors of post-transplant pneumonia [5]. Thus, PRCs after transplantation negatively impact mortality [3], [4], [6], with recent data showing a mortality rate of 40% [9].

Noninvasive ventilation (NIV) is an effective treatment for acute respiratory failure in many conditions [10]–[14]. Two randomized controlled studies showed its effectiveness in patients with acute respiratory failure under immunosuppressed conditions [11], [14]. On the other hand, in immunosuppressed patients who failed NIV, the rate of hospital mortality was reported to be very high, ranging from 62% to 100% [11], [15], [16]. Recent data on patients with hematologic malignancies showed that the reintubation rate with NIV was almost 50% and that the mortality rate following NIV failure amounted to 69–79% [15], [17]. The respiratory rate during NIV and a longer delay between admission and its first use of NIV as well as other factors were significantly associated with NIV failure [15]. However, factors related to reintubation following NIV for patients with PRCs after LT have not been documented well. In our hospital, we have successfully begun to apply NIV for respiratory complications in patients with living donor liver transplantation (LDLT) [18]–[21]. Although we have subsequently experienced many more cases (over 200 cases) for whom NIV was used following LDLT, reintubation has been necessary in some of these patients. Therefore, to decrease the rate of reintubation following NIV treatment after LDLT and to achieve a better prognosis, we have retrospectively examined patient data to elucidate the factors necessitating reintubation following NIV treatment. We also compared clinical outcomes between patients who did and did not require reintubation after NIV treatment following LT.

## Methods

### Patients

From August 1999 to July 2008, 532 liver transplant recipients, aged 13 years or over, underwent LDLT at Kyoto University Hospital. Of the 200 patients who subsequently received NIV, we excluded 21 who discontinued NIV therapy because of reoperation (regardless of their respiratory status) and analyzed data on the remaining 179 patients. Fifteen of the 179 patients had infections that could be expected to be successfully treated before LT but that did result in postponement of the LT. These patients received LT after the infections were controlled as evidenced by reduced fever, blood cultures negative for bacteria, and resolution of conditions such as pneumonia, peritonitis, cholangitis, phlegmon, or enterocolitis.

After LDLT, all patients entered the Intensive Care Unit (ICU) and required invasive mechanical ventilation before weaning. Extubation was considered under the following conditions: 1) clinically stability; 2) improvement in underlying disease and its complications had improved; 3) minimal ventilator support was minimal (pressure support <6 cm H_2_O with positive end-expiratory pressure (PEEP) ≤4 cm H_2_O); and 4) sufficient spontaneous breathing was sufficient. The Ethics Committee of Kyoto University gave approval for the protocol of this study.

### Introduction of NIV

NIV was considered for all patients who received oxygen therapy or were in case of reintubation and mechanical ventilation and who met at least one of the following criteria to indicate serious PRCs: 1) ratio of the partial pressure of arterial oxygen (PaO_2_) to the fraction of inspired oxygen (F_I_O_2_) (PaO_2_/F_I_O_2_)≤250 while the patient was receiving oxygen therapy; 2) partial pressure of arterial carbon dioxide (PaCO_2_) ≥45 Torr; 3) presence of pneumonia while on oxygen therapy; 4) respiratory rate >25 breaths per minute with active contraction of accessory muscles of respiration and/or paradoxical thoraco-abdominal motion; 5) atelectasis of more than one lobe; 6) massive or uncontrolled pleural effusion after percutaneous thoracic drainage, because some of the effusion might be ascites from the abdomen by the pressure gradient [22] ; and 7) other reasons. F_I_O_2_ of oxygen therapy via a nasal cannula, face mask, or reservoir face mask was calculated based on a previously published method [23]. Patients who required urgent intubation due to respiratory arrest, respiratory pauses, severe hepatic coma (above Grade 2), copious tracheal secretion and hemodynamic instability were not started on NIV.

### Noninvasive Ventilation

We used a full-face mask or a nasal mask (Resmed, North Ryde, New South Wales, Australia) for NIV. Ventilation in all patients was by bilevel positive airway pressure (bilevel PAP) devices with oxygen and humidification (VPAP series Resmed) [18]–[21], [24], [25]. After the mask had been secured, the level of support pressure and expiratory positive airway pressure (EPAP) and the amount of oxygen were progressively increased until SaO_2_ was >95%, accompanied by decreased respiratory rates and/or reduced activity of accessory muscles for respiration, decreased paradoxical thoraco-abdominal movement, and improvement in respiratory discomfort. When applying NIV, a doctor stayed at the bedside and observed the patient carefully while the SaO_2_ and electrocardiogram were monitored. Throughout the first hour, the patient's condition was assessed repeatedly. For minor complications of NIV treatment such as skin rash, eye irritation, discomfort from the mask, air pressure, or gastric insufflations, we decreased the pressure and/or usage time of NIV, used another mask, or inserted a gastric tube. To calculate the F_I_O_2_ during NIV, we used the value from the information supplied by the manufacturer and was attached to the mask. Using this information, the F_I_O_2_ was determined from the following parameters: leakage flow rate per minute from the mask at each pressure and the oxygen flow rate per minute during NIV. If the leakage flow rate at the setting was X and the oxygen flow rate was Y, F_I_O_2_ at the setting was: F_I_O_2_ = {Y×1.0+(X−Y)×0.21}/X [20].

### Discontinuation of NIV

Patients for whom NIV could be discontinued because their respiratory status (including chest X-ray abnormality) had improved were assigned to the success group. The reintubated group was comprised of patients for whom NIV was not successful and who underwent reintubation with mechanical ventilation were assigned to the reintubated group. Criteria for reintubation were as follows; failure to maintain SaO_2_ of >90% with a F_I_O_2_ ≥0.6; development of conditions necessitating endotracheal intubation to protect the upper airway (seizure, severe hepatic coma); development of copious tracheal secretions that could not be expectorated; increase in the PaCO_2_ accompanied by a pH of ≤7.30; and severe hemodynamic instability defined as systolic blood pressure <70 mmHg.

### Data Collection

Pneumonia was defined as new onset of pulmonary infiltrates with clinical symptoms (fever, cough, purulent tracheobronchial secretions, and dyspnea at rest), leukocytosis, and detection of potentially pathogenic bacteria in the sputum or bronchoalveolar lavage culture. Other infectious complications were wound infection, liver abscess, subphrenic abscess, cholangitis, peritonitis, and urinary tract infection. These were confirmed by clinical observation (fever, purulent discharge from wound, abdominal pain), and laboratory markers of inflammation with positive cultures (blood, bile, pus, and urine), and findings from chest X-rays and/or chest computed tomography. The Acute Physiology and Chronic Health Evaluation (APACHE) II score was used to assess the severity of illness at ICU admission [26]. Postoperative laboratory data presented in Table 1 represent values that were obtained on the morning of the introduction of NIV. Arterial blood gases were obtained before the introduction of NIV, and also at the initial assessment after applying NIV (mean time ± standard deviation (SD) following NIV introduction: 3.9±4.4 hours). At the initial assessment after NIV, we could not obtain arterial blood gas in 13 of the 179 patients (7.3%).

**Table 1:** Operative and postoperative status of 179 recipients of NIV.

	Overall (n = 179)	Reintubation (n = 43)	NIV Success (n = 136)	p value
ABO Incomptible	45 (25.1)	17 (39.5)	28 (20.6)	0.01
APACHE II	16.4±4.3	18.5±4.8	15.7±3.9	0.0001
Postoperative data:				
Hb (g/dl)	9.4±1.8	8.7±1.6	9.6±1.8	0.005
Total bilirubin (mg/dl)	7.3±7.2	11.8±9.4	5.8±5.7	<0.0001
CRP (mg/dl)	4.3±3.6	5.6±4.0	3.9±3.4	0.005
Na(mEq/l)	135.7±4.6	134.1±4.4	136.1±4.6	0.01
HR (beats per minute)	92±18	100±19	89±17	0.0009
RR (beats per minute)	19±8	22±8	18±7	0.008
Reintubation before NIV	19 (10.6)	9 (20.9)	10 (7.4)	0.02
Extubation (days)	3.4±5.3	6.6±8.1	2.4±3.5	<0.0001
From Extubation to NIV(days)	2.7±7.7	2.9±7.9	2.6±6.3	0.80
Reasons for NIV:				
PaO_2_/F_I_O_2_ ≤250	95 (53.1)	20 (46.5)	75 (55.1)	0.32
PaCO_2_ ≥45 Torr	40 (22.3)	9 (20.9)	31 (22.8)	0.80
Pneumonia on NIV	24 (13.4)	12 (27.9)	12 (8.8)	0.001
Respiratory rate ≥25/min	28 (15.6)	11 (25.6)	17 (12.5)	0.04
Atelectasis	31 (17.3)	4 (9.3)	27 (19.9)	0.11
Massive pleural effusion	96 (53.6)	25 (58.1)	71 (52.2)	0.50
Other reasons	27 (15.1)	8 (18.6)	19 (14.0)	0.46
Settings of NIV:				
Mode (S/T/ST)	1/1/177	0/1/42	1/0/135	0.17
IPAP (cmH_2_O)	8.8±1.5	9.0±1.6	8.7±1.5	0.26
EPAP (cmH_2_O)	4.6±1.3	4.2±0.5	4.3±0.6	0.54
Amount of oxygen (l/min)	8.8±3.4	9.7±3.2	8.5±3.4	0.04

mean ± SD or number (%) Abbreviations: APACHE II, Acute Physiology and Chronic Health Evaluation II; Hb, hemoglobin; CRP, C reactive protein; Na, sodium; HR, heart rate; RR, respiratory rate; NIV, noninvasive ventilation; LDLT, living-donor liver transplantation; PaO_2_, partial pressure of arterial oxygen; F_I_O_2_, fraction of inspired oxygen; PaCO_2_, partial pressure of arterial carbon dioxide; S, spontaneous; T, timed; ST, spontaneous and timed; IPAP, inspiratory positive airway pressure; EPAP, expiratory positive airway pressure.

### Statistical Analysis

Data were analyzed using JMP 9.0 (SAS Institute, Inc. Cary, NC, USA), and values are expressed as mean ± SD or absolute numbers and percentages in each group. We compared the association between the perioperative factors and the results of NIV (success group or reintubated group). Continuous variables were tested by the unpaired t test or Mann-Whitney U test. Categorical variables were compared using the χ^2^ test or the Fisher's exact test. A p value <0.05 was considered to indicate statistical significance. Next, we investigated the associations between perioperative factors and reitubation. Possible predictors of reintubation were tested by univariate and multivariate logistic regression analysis. In the logistic regression analysis for reintubation, variables entered in the multivariate analysis were those yielding a p value <0.05 by univariate analysis; p values <0.05 in the multivariate analysis were considered statistically significant.

## Results

### Preoperative and Postoperative Characteristics of the Patients with NIV

The preoperative characteristics and operative and postoperative status of the 179 recipients of NIV are summarized in Tables 1 and 2, respectively. The mean model for end-stage liver disease (MELD) score was 24.2±11.0 in the 179 patients. Fifteen patients had controlled preoperative infections; 5 pneumonia, 7 spontaneous bacterial peritonitis (SBP), and 1 either cholangitis, phlegmon, or enterocolitis. As mentioned above, these preoperative infections had been controlled before the LT (controlled preoperative infections) (Table 2). Before NIV treatment, 19 (10.6%) of the 179 patients had been reintubated following the LT for the following reasons: copious amounts of sputum that could not be expectorated, septic shock, pneumonia, tracheal hemorrhage, and respiratory muscle fatigue. NIV was introduced following the second extubation in these 19 patients (Table 1).

**Table 2:** Preoperative characteristics of 179 recipients of NIV.

	Overall (n = 179)	Reintubation (n = 43)	NIV Success (n = 136)	p value
Male	92 (51.4)	19 (44.2)	73 (53.7)	0.28
Age (years)	48.2±13.2	48.7±12.5	44.3±15.7	0.19
Underlying disease:				0.13
Hepatitis B or C, PBC,				
Fulminant hepatitis and others				
Preoperative status:				
Residence in ICU with intubation before LT	23 (12.8)	11 (25.6)	12 (8.8)	0.004
Child-Pugh (points)	10.3±2.0	10.8±2.0	10.1±1.9	0.06
MELD score	24.2±11.0	28.1±12.1	22.9±10.3	0.006
Chest X-ray abnormality:	37 (20.7)	13 (30.2)	24 (17.6)	0.08
Controlled pre-OP infections	15 (8.4)	8 (18.6)	7 (5.1)	0.01

mean ± SD or number (%).

Abbreviations: ICU, Intensive Care Unit; LT, liver transplantation; MELD, model for end-stage liver disease; pre-OP, preoperative.

### Noninvasive Ventilation

Reasons for application of NIV are listed in Table 1. Concerning the duration of the initial application of NIV, 137 patients (87.3%) received NIV continuously throughout the day, 17 (10.8%) 2 or 3 times per day, 1–2 hours per NIV session, and 3 (1.9%) only nocturnally. In 43 patients (24.0%), reintubation following NIV was required for the following reasons: refractory hypoxemia with pneumonia (n = 17, 39.5%), acute respiratory distress syndrome (ARDS) (n = 2, 4.7%), secretions that could not be cleared (n = 12, 27.9%), unconsciousness (n = 6, 14.0%), septic shock (n = 2), CO_2_ narcosis (n = 1, 2.3%), excessive tachypnea (n = 1), re-expansion pulmonary edema (n = 1), and rupture of an esophageal varix (n = 1).

### Outcomes of Patients with NIV Treatment

Table 3 shows the outcome of the NIV treatment. No evidence of an obvious delay in reintubation was noted, and no severe complications such as pneumothorax, hypotension, or aspiration pneumonia were related to NIV treatment.

**Table 3:** Outcome of 179 hospitalized recipients of NIV.

	Overall (n = 179)	Reintubation (n = 43)	NIV Success (n = 136)	p value
PaO_2_/F_I_O_2_ before NIV	255±114	263±125	252±110	0.60
PaO_2_/F_I_O_2_ after NIV	328±117	301±120	338±115	0.07
PaCO_2_ before NIV	41±7	41±7	42±7	0.55
PaCO_2_ after NIV	41±6	41±7	42±6	0.33
NIV intolerant	16 (8.9)	7 (16.3)	9 (6.6)	0.06
Could not tolerate NIV	9 (5.0)	5 (11.6)	4 (2.9)	0.04
Suspended NIV due to complications	7 (3.9)	2 (4.7)	5 (3.7)	0.99
Hospital mortality, due to:	30 (16.8)	22 (51.2)	8 (5.9)	<0.0001
Respiratory complications	14 (7.8)	11 (25.6)	3 (2.2)	<0.0001
Pneumonia	9 (5.0)	8 (18.6)	1 (0.7)	<0.0001
Aspergillosis	3 (1.7)	2 (4.7)	1 (0.7)	0.56
Hemorrhage	2 (1.1)	1 (2.3)	1 (0.7)	0.42
Others:	16 (8.9)	11 (25.6)	5 (3.7)	<0.0001
Graft failure	6 (3.4)	2 (4.7)	4 (2.9)	0.63
Cerebral diseases	2 (1.1)	1 (2.3)	1 (0.7)	0.42
Sepsis	4 (2.2)	4 (9.3)	0	0.003
Gastrointestinal bleeding	4 (2.2)	4 (9.3)	0	0.003
Hospitalization (days)	75.5±63.8	103.6±98.0	66.7±45.2	0.0008
ICU stay (days)	9.2±11.1	19.7±15.7	5.9±6.4	<0.0001
Duration of NIV (days)	13.4±14.3	6.3±6.6	15.6±15.3	0.0001
Postoperative infections	89 (49.7)	36 (83.8)	53 (39.0)	<0.0001
Respiratory	47 (26.3)	32 (74.4)	15 (11.0)	<0.0001
Others	66 (36.7)	21 (48.8)	45 (33.1)	0.06
Reoperation	49 (27.3)	21 (48.8)	28 (20.6)	0.0003
HAT	5 (2.8)	2 (4.7)	3 (2.2)	0.60
Biliary leak	12 (6.7)	4 (9.3)	8 (5.9)	0.49
Acute cellular rejection	28 (15.6)	6 (14.0)	22 (16.2)	0.81
Ileus	4 (2.2)	0	4 (2.9)	0.57
ARF after LT	15 (8.4)	5 (11.6)	10 (7.4)	0.36

mean ± SD or number (%).

Abbreviations: NIV, noninvasive ventilation; PaO_2_, partial pressure of arterial oxygen; F_I_O_2_, fraction of inspired oxygen; PaCO_2_, partial pressure of arterial carbon dioxide; NIV, noninvasive ventilation; ARDS, Acute Respiratory Distress Syndrome; ICU, Intensive Care Unit; HAT, hepatic artery thrombosis; ARF, acute renal failure; LT, liver transplantation.

In both the success and reintubation groups, the baseline PaO_2_/F_I_O_2_ values were similar and the PaO_2_/F_I_O_2_ at the initial assessment after NIV therapy was higher in the success group than in the reintubation group but without significance (p = 0.07) (Table 3). However, a sub-analysis showed that in patients with pneumonia prior to application of NIV, the baseline PaO_2_/F_I_O_2_ was similar between groups (success group: n = 12, 284.4±118.2 vs. reintubation group: n = 12, 231.7±163.0, p = 0.37), whereas PaO_2_/F_I_O_2_ at the initial assessment after NIV therapy was higher in the success group than in the reintubation group (success group: n = 12, 376.8±140.1 vs. reintubation group: n = 12, 263.6±129.4, p = 0.04).

Although there was no significant between-group differences in the mean changes in PaCO_2_ at the initial assessment after the start of NIV therapy (Table 3), PaCO_2_ levels after NIV treatment significantly decreased in patients with PaCO_2_ ≥45 Torr (success group: n = 27, 51.0±4.5 Torr to 48.8±5.3 Torr, p = 0.008; reintubation group: n = 7, 52.3±7.2 Torr to 48.4±5.6 Torr, p = 0.02).

Eight (5.9%) of the 136 patients in whom NIV was successful died during hospitalization, while 22 (51.2%) of the 43 patients who failed NIV treatment died (p<0.0001). NIV treatment could not be continued in 16 patients for various reasons. In 7 of the 16 patients, NIV was suspended due to complications (6 severe abdominal distension despite a nasal gastric tube; 1 concomitant ileus). Nine patients could not tolerate NIV, and the prevalence of those who could not tolerate NIV was significantly higher in reintubation group than in NIV success group (Table 3). Among the 16 patients in whom NIV discontinued, 7 were eventually reintubated and 5 of those 7 died.

**Figure 1:**
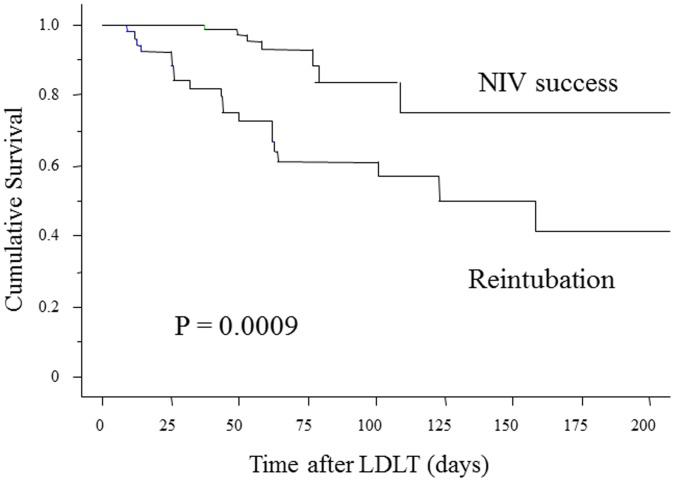
Survival curve following LDLT in NIV success group and reintubation group. Patients who failed NIV had a significantly poorer prognosis (p = 0.0009). Abbreviations: LDLT, living-donor liver transplantation; NIV, noninvasive ventilation.

The survival curve shows that patients in the reintubation group had a significantly poorer prognosis than those in the NIV success group (p = 0.0009) (Figure 1). Also, patients who failed NIV had significant longer ICU stays (19.7±15.7 days vs. 5.9±6.4, p<0.0001).

### Logistic Regression Analysis for reintubation

Among the 40 perioperative factors (those listed in Tables 1 and 2 and NIV intolerance), 16 had a significant association with reintubation in the univariate analysis (Table 4). In the multivariate forward logistic analysis of factors related to reintubation, controlled preoperative infections (odds ratio [OR] 8.88; 95% confidence interval (CI) 1.64 to 48.11; p = 0.01), ABO-incompatibility (OR 4.49; 95% CI, 1.50 to 13.38; p = 0.007), and postoperative pneumonia prior to starting NIV (OR 3.28; 95% CI, 1.02 to 11.01; p = 0.04) were significant risk factors for reintubation (Table 5). The R square in this model was 0.299. If the 19 patients who were administered NIV following the second extubation were excluded from the study, the same three conditions were also significant risk factors for reintubation: controlled preoperative infections (odds ratio [OR] 7.12; 95% confidence interval (CI) 1.46 to 34.81; p = 0.02), ABO-incompatibility (OR 3.49; 95% CI, 1.03 to 11.83; p = 0.04), and postoperative pneumonia prior to starting NIV (OR 3.75; 95% CI, 1.12 to 12.56; p = 0.03).

**Table 4:** Univariate analysis of factors related to reintubation.

	OR	95% CI	p value
Preoperative status			
Residence in ICU with intubation before LDLT	3.6	1.4 to 8.8	0.006
MELD score	1.0	1.0 to 1.1	0.008
Comorbidity			
Controlled preoperative infectious disease	4.2	1.4 to 12.4	0.009
Operative status			
ABO compatibility			
Incompatible	2.5	1.2 to 5.3	0.01
Postoperative status			
APACHE II	1.2	1.1 to 1.3	0.0004
Hb	0.7	0.6 to 0.9	0.007
Serum total bilirubin	1.1	1.1 to 1.2	<0.0001
Serum CRP	1.1	1.0 to 1.2	0.008
Serum Na	0.9	0.8 to 1.0	0.02
HR	1.0	1.0 to 1.1	0.002
RR	1.1	1.0 to 1.1	0.01
Reintubation before NIV	3.3	1.3 to 8.9	0.02
Extubation days	1.2	1.1 to 1.9	0.0006
Criteria for NIV			
Postoperative pneumonia on NIV	4.0	1.6 to 9.8	0.002
Respiratory rate ≥25 breaths/min	2.4	1.0 to 5.7	0.04
NIV setting			
Oxygen flow (l/min)	1.1	1.0 to 1.2	0.04

Abbreviations: OR, Odds ratio; 95%CI, 95% Confidence Interval; ICU, Intensive Care Unit; LDLT, living-donor liver transplantation; MELD, model for end-stage liver disease; APACHE II, Acute Physiology and Chronic Health Evaluation II; Hb, hemoglobin; CRP, C reactive protein; Na, sodium; HR, heart rate; RR, respiratory rate; NIV, noninvasive ventilation.

**Table 5:** Multivariate forward logistic regression analysis for reintubation.

	OR	95% CI	p value	
Controlled preoperative infectious disease	8.9	1.6 to 48.1	0.01	
ABO compatibility: Incompatible	4.5	1.5 to 13.4	0.007	
Postoperative pneumonia on NIV	3.3	1.0 to 11.0	0.04	
R square of the model				0.299

Abbreviations: OR, Odds ratio; 95%CI, 95% Confidence Interval; NIV, noninvasive ventilation.

## Discussion

Although the data were retrospective, this study of a large NIV series in one hospital showed the following significant factors that were predictive of reintubation following NIV: controlled preoperative infections, ABO blood type incompatibility, and postoperative pneumonia prior to placement on NIV. After excluding from the analysis the 19 patients who were reintubated following LDLT and were provided NIV after that second extubation, those three factors remained significant. In addition, after the start of NIV treatment in pneumonia patients, there was a significant difference not in the initial PaO_2_/F_I_O_2_ but in the initial assessments of PaO_2_/F_I_O_2_ between the reintubation and success groups.

Patients who are waiting for transplantation are usually have severely ill and are sometimes immunocompromised. Therefore, infections develop easily, which often postpones the transplantation. In this study, 15 (8.4%) of the 179 patients who had been administered NIV had an infection that had been controlled before LT. Although the LT team considered that the preoperative infection had been well controlled, this factor was revealed to be one of 3 factors predictive of reintubation following NIV. Since the number of patients with a preoperative infection (n = 15) was small, it was difficult to make firm conclusions as to the role of preoperative infections in the failure of NIV. In addition to control of preoperative infections as stringent as possible by LDLT teams, the future study of this issue in a greater number of patients should be done.

Prognosis of ABO-incompatible LT in adults has been reported to be inferior to compatible LT because of rejection, and especially there has been a high incidence of acute bile duct and vascular complications [27]. Although it is difficult to determine the contributions of ABO-incompatible LT to reintubation following NIV, results of this study suggest that patients with NIV following ABO-incompatible LT should be cautiously observed for PRCs, which might be influenced by several complications due to an ABO-incompatible LT.

A report from hematological parts showed that NIV treatment for respiratory failure with acute lung injury (ALI) or ARDS had a high mortality rate [17]. In the present report, the number of patients with ALI/ARDS was small and pneumonia prior to NIV treatment following LT was a significant risk factor for reintubation. In the success group and reintubated patients with pneumonia prior to NIV, the baseline PaO_2_/F_I_O_2_ was similar, whereas PaO_2_/F_I_O_2_ at the initial assessment after NIV therapy was higher in the success group than in the reintubation group. Pneumonia was already identified as a risk factor for NIV failure [28]. Therefore, we propose that if the PaO_2_/F_I_O_2_ does not improve in patients with pneumonia after application of NIV, reintubation should be performed early. However in this study, PaO_2_/F_I_O_2_ was not true but the calculated ones from the formula [20]. Therefore, the findings on PaO_2_/F_I_O_2_ in pneumonia patients in this study were not conclusive and this issue requires further study.

Our inclusion in the analysis of the 19 patients who were reintubated following LDLT and were then provided NIV after their second extubation might be questioned as those patients could be considered to represent a separate group of NIV post-transplant recipients. However, we included these patients in the overall analysis because we wanted to provide information for clinicians on all of our patients who received NIV treatment following LDLT. To determine if risk factors for reintubation following LDLT differed among these 19 patients from those in the overall study group, we performed a separate analysis and found that the same three risk factors existed for these patients as for the overall study group.

In our study, 16 patients were NIV intolerant. Seven of these patients were eventually reintubated and 5 of the 7 died. Ambrosino et al. reported that intolerance to NIV treatment was associated with NIV failure [29]. Thus, new equipment such new types of masks [30], [31] or new types of machines that alleviate the discomfort due to ventilator-related pressure or flow might be helpful to decrease the rate of intolerance to NIV.

Recently, the first review of NIV in adult liver transplantation was published [32]. Although this report was comprehensive regarding the usefulness of NIV during the perioperative LDLT stage, risk factors for reintubation following NIV treatment were not discussed, which is the topic of the current report. Previously, although we addressed the general effectiveness of NIV in both adult and infant patients [18]–[21], [24], [25], this report provides more specific information on the topic than those reports or the recent review. This retrospective study was done in an institution with extensive experience in the use of NIV [2], and the relatively high rate of use of NIV treatment could be explained by the high mean MELD scores (24.2) in the 179 patients whose data were analyzed. Although these study patients already had relatively severe morbidity before they underwent LDLT, the rate of NIV success was 76.0%. This rate was higher or equivalent to that in immunosuppressed patients in previous reports [10], [13], [14]. In our institution, we set relatively mild inclusion criteria for introducing NIV, and started NIV early, partly because non-infectious respiratory complications in the patients following liver transplantation were reported as the independent risk factors of pneumonia [5] and respiratory failure might deteriorate rapidly in immunosuppressed patients after LDLT. The early introduction of NIV might result in the higher the rate of NIV success and lower hospital mortality rate.

This study had several limitations. Firstly, was the retrospective design. However, the large number of cases included in our analysis was probably sufficient to minimize this limitation. Secondly, based on several factors present in the perioperative stage, patients in the reintubated group had a more serious condition than those in the NIV success group. Therefore, success or failure might be dependent on the patients' condition before NIV treatment. It is difficult to know how these conditions before NIV treatment influenced the success or failure of the NIV treatment. These complicated backgrounds might have caused the R square in the multivariate forward logistic analysis for reintubation to be comparatively low (Table 5). However, it is important to manage NIV treatment so that success is achieved without its overuse.

In conclusion, we demonstrated that controlled preoperative infections, ABO blood type incompatibility, and postoperative pneumonia prior to the start of NIV were early predictors of NIV failure. We also revealed that in patients with postoperative pneumonia being administered NIV, PaO_2_/F_I_O_2_ at the initial assessment after NIV therapy was higher in the success group than in the reintubation group. We propose that if patients with a preoperative infection, ABO-incompatibility or post-operative pneumonia receiving NIV do not show improvement in the PaO_2_/F_I_O_2_ after NIV, early endotracheal intubation with mechanical ventilation should be considered as an alternative therapy.

## References

[1] Müllhaupt B, Dimitroulis D, Gerlach JT, Clavien PA (2008) Hot topics in liver transplantation: organ allocation–extended criteria donor--living donor liver transplantation. J Hepatol 48: S58–67. 18308415 10.1016/j.jhep.2008.01.013

[2] Tanaka K, Ogura Y, Kiuchi T, Inomata Y, Uemoto S, et al (2004) Living donor liver transplantation: Eastern experiences. HPB (Oxford) 6: 88–94. 18333056 10.1080/13651820310020765 PMC2020667

[3] Singh N, Gayowski T, Wagener MM, Marino IR (1999) Pulmonary infiltrates in liver transplant recipients in the intensive care unit. Transplantation 67: 1138–44. 10232564 10.1097/00007890-199904270-00009

[4] Hasegawa S, Mori K, Inomata Y, Murakawa M, Yamaoka Y, et al (1996) Factors associated with postoperative respiratory complications in pediatric liver transplantation from living-related donors. Transplantation 62: 943–7. 8878388 10.1097/00007890-199610150-00012

[5] Golfieri R, Giampalma E, Morselli Labate AM, d'Arienzo P, Jovine E, et al (2000) Pulmonary complications of liver transplantation: radiological appearance and statistical evaluation of risk factors in 300 cases. Eur Radiol 10: 1169–83. 11003416 10.1007/s003309900268 PMC7102073

[6] Durán FG, Piqueras B, Romero M, Carneros JA, de Diego A, et al (1998) Pulmonary complications following orthotopic liver transplant. Transpl Int 11: S255–9. 9664991 10.1007/s001470050473

[7] Hong SK, Hwang S, Lee SG, Lee LS, Ahn CS, et al (2006) Pulmonary complications following adult liver transplantation. Transplant Proc 38: 2979–81. 17112879 10.1016/j.transproceed.2006.08.090

[8] Lin CC, Chuang FR, Wang CC, Chen YS, Chen CL, et al (2004) Early postoperative complications in recipients of living donor liver transplantation. Transplant Proc 36: 2338–41. 15561242 10.1016/j.transproceed.2004.07.044

[9] Huang CT, Lin HC, Chang SC, Lee WC (2011) Pre-operative risk factors predict post-operative respiratory failure after liver transplantation. PLoS One 6: e22689. 21829646 10.1371/journal.pone.0022689 PMC3148242

[10] Ferrer M, Esquinas A, Leon M, Gonzalez G, Alarcon A, et al (2003) Noninvasive ventilation in severe hypoxemic respiratory failure: a randomized clinical trial. Am J Respir Crit Care Med 168: 1438–44. 14500259 10.1164/rccm.200301-072OC

[11] Hilbert G, Gruson D, Vargas F, Valentino R, Gbikpi-Benissan G, et al (2001) Noninvasive ventilation in immunosuppressed patients with pulmonary infiltrates, fever, and acute respiratory failure. N Engl J Med 344: 481–7. 11172189 10.1056/NEJM200102153440703

[12] Nava S, Hill N (2009) Non-invasive ventilation in acute respiratory failure. Lancet 374: 250–9. 19616722 10.1016/S0140-6736(09)60496-7 PMC7138083

[13] Ambrosino N, Vagheggini G (2008) Noninvasive positive pressure ventilation in the acute care setting: where are we? Eur Respir J 31: 874–86. 18378782 10.1183/09031936.00143507

[14] Antonelli M, Conti G, Bufi M, Costa MG, Lappa A, et al (2000) Noninvasive ventilation for treatment of acute respiratory failure in patients undergoing solid organ transplantation: a randomized trial. JAMA 283: 235–41. 10634340 10.1001/jama.283.2.235

[15] Adda M, Coquet I, Darmon M, Thiery G, Schlemmer B, et al (2008) Predictors of noninvasive ventilation failure in patients with hematologic malignancy and acute respiratory failure. Crit Care Med 36: 2766–72. 18766110 10.1097/CCM.0b013e31818699f6

[16] Confalonieri M, Calderini E, Terraciano S, Chidini G, Celeste E, et al (2002) Noninvasive ventilation for treating acute respiratory failure in AIDS patients with Pneumocystis carinii pneumonia. Intensive Care Med 28: 1233–8. 12209270 10.1007/s00134-002-1395-2

[17] Gristina GR, Antonelli M, Conti G, Ciarlone A, Rogante S, et al (2011) Noninvasive versus invasive ventilation for acute respiratory failure in patients with hematologic malignancies: a 5-year multicenter observational survey. Crit Care Med 39: 2232–9. 21666446 10.1097/CCM.0b013e3182227a27

[18] Chin K, Uemoto S, Takahashi K, Egawa H, Kasahara M, et al (2005) Noninvasive ventilation for pediatric patients including those under 1-year-old undergoing liver transplantation. Liver Transpl 11: 188–95. 15666379 10.1002/lt.20297

[19] Murase K, Chihara Y, Takahashi K, Okamoto S, Segawa H, et al (2012) The use of noninvasive ventilation for pediatric patients following liver transplantation: Decrease in the need for reintubation. Liver Transpl 18: 1217–25. 22692821 10.1002/lt.23491

[20] Chihara Y, Egawa H, Tsuboi T, Oga T, Handa T, et al (2011) Immediate Noninvasive Ventilation May Improve Mortality in Patients with Hepatopulmonary Syndrome Following Liver Transplantation. Liver Transpl 17: 144–8. 21280187 10.1002/lt.22207

[21] Takahashi K, Chin K, Ogawa K, Kasahara M, Sakaguchi T, et al (2005) Living donor liver transplantation with noninvasive ventilation for exertional heat stroke and severe rhabdomyolysis. Liver Transpl 11: 570–2. 15838872 10.1002/lt.20400

[22] Takahashi K, Chin K, Sumi K, Nakamura T, Matsumoto H, et al (2005) Resistant hepatic hydrothorax: a successful case with treatment by nCPAP. Respir Med 99: 262–4. 15733499 10.1016/j.rmed.2004.08.001

[23] Barry A, Shapiro B (1985) Clinical application of respiratory care. Third edition. Mosby 180–187.

[24] Chin K, Takahashi K, Ohmori K, Toru I, Matsumoto H, et al (2007) Noninvasive ventilation for pediatric patients under 1 year of age after cardiac surgery. J Thorac Cardiovasc Surg 134: 260–1. 17599530 10.1016/j.jtcvs.2007.03.002

[25] Narita M, Hatano E, Nagata H, Yanagida A, Asechi H, et al (2009) Prophylactic respiratory management after liver resection with bilevel positive airway pressure ventilation: Report of three cases. Surg Today 39: 172–4. 19199000 10.1007/s00595-008-3815-6

[26] Knaus WA, Draper EA, Wagner DP, Zimmerman JE (1985) APACHE II: a severity of disease classification system. Crit Care Med 13: 818–29. 3928249

[27] Wu J, Ye S, Xu X, Xie H, Zhou L, et al (2011) Recipient outcomes after ABO-incompatible liver transplantation: a systematic review and meta-analysis. PLoS One 6: e16521. 21283553 10.1371/journal.pone.0016521 PMC3026838

[28] Antonelli M, Conti G, Moro ML, Esquinas A, Gonzalez-Diaz G, et al (2001) Predictors of failure of noninvasive positive pressure ventilation in patients with acute hypoxemic respiratory failure: a multi-center study. Intensive Care Med 7: 1718–28. 10.1007/s00134-001-1114-4 11810114

[29] Ambrosino N, Foglio K, Rubini F, Clini E, Nava S, et al (1995) Non-invasive mechanical ventilation in acute respiratory failure due to chronic obstructive pulmonary disease: correlates for success. Thorax 50: 755–7. 7570410 10.1136/thx.50.7.755 PMC474648

[30] Antonelli M, Conti G, Pelosi P, Gregoretti C, Pennisi MA, et al (2002) New treatment of acute hypoxemic respiratory failure: noninvasive pressure support ventilation delivered by helmet–a pilot controlled trial. Crit Care Med 30: 602–8. 11990923 10.1097/00003246-200203000-00019

[31] Rocco M, Dell'Utri D, Morelli A, Spadetta G, Conti G, et al (2004) Noninvasive ventilation by helmet or face mask in immunocompromised patients: a case-control study. Chest 126: 1508–15. 15539720 10.1378/chest.126.5.1508

[32] Feltracco P, Serra E, Barbieri S, Milevoj M, Salvaterra F, et al (2008) Noninvasive ventilation in adult liver transplantation. Transplant Proc 40: 1979–82. 18675106 10.1016/j.transproceed.2008.05.006

